# Medical haematology: Repositioning haematology at the centre of medicine

**DOI:** 10.1111/bjh.70340

**Published:** 2026-01-20

**Authors:** Cheng Hock Toh, Imelda Bates, Sue Pavord

**Affiliations:** ^1^ Department of Clinical Infection, Microbiology and Immunology University of Liverpool Liverpool UK; ^2^ Department of Haematology University Hospitals of Liverpool Group Liverpool UK; ^3^ Centre for Capacity Research Liverpool School of Tropical Medicine Liverpool UK; ^4^ Department of Haematology Oxford University Hospitals NHS Foundation Trust Oxford UK

**Keywords:** anaemia, education, medical haematology, non‐malignant haematology, research, thrombosis, workforce

## Abstract

Haematology is at a crossroads, divided between haemato‐oncology and the disparate disciplines collectively known as ‘non‐malignant’ haematology. This latter term is a misnomer that devalues a spectrum of complex, life‐threatening conditions and contributes to workforce shortages and research inequities. This article argues for the formal adoption of the term Medical Haematology to redefine this domain. We chart its central role across medicine, from guiding anticoagulation, transfusion and thrombosis care across specialties to addressing global health challenges. We highlight its pioneering contributions to molecular medicine and immunotherapy, exemplified by gene therapy for haemophilia and the repurposing of chimeric antigen receptor T cells for autoimmune disease. Finally, we present a forward‐looking blueprint involving establishment of ‘Blood Teams’, revamping educational curricula and championing equity to secure the speciality's future. Embracing Medical Haematology is a strategic imperative to reflect the life‐threatening nature of many conditions within the speciality, attract trainees, rebalance research priorities and firmly re‐establish haematology's indispensable role at the heart of modern medical practice.

## Graphical abstract

The complementary domains of Medical Haematology and Haemato‐Oncology share a biological framework. The left panel depicts red cells, platelets, coagulation, thrombosis and innate immunity, while the right panel shows lymphoid and myeloid malignancies. A central DNA helix highlights common molecular and translational pathways across the speciality of haematology.

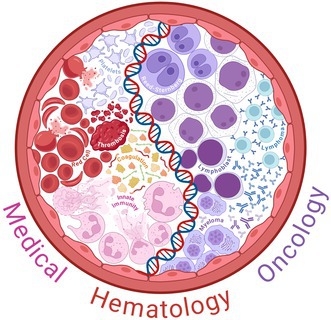

## INTRODUCTION

Since antiquity, the study of blood and its disorders has been a cornerstone of medical practice. From Hippocrates' humoral theory to the modern era of gene editing and immunotherapy, the field of haematology has consistently been a driver of medical progress.^1^ The central role of blood in human physiology and disease has put the field at the forefront of advances in medicine.

Over recent years, haematology has seen an increasing divide between malignant haematology, which focuses on blood cancers such as leukaemia, lymphoma, myeloma and related stem cell transplantation, and the rest that has by default become collectively referred to as non‐malignant haematology. The latter, also referred to as benign or classical haematology, encompasses both primary haematological conditions, such as sickle cell disease, thrombotic thrombocytopenic purpura (TTP) and haemophilia, and secondary conditions such as heparin‐induced thrombocytopenia, obstetric haemorrhage and sepsis‐induced disseminated intravascular coagulation. The aggressive and life‐threatening nature of many of these diseases shows that the term non‐malignant is misleading.^2^

Indeed, this aspect of haematology plays an essential part in the management of hospitalised patients—if not an overt role, then one in the background, such as ensuring quality and interpretation of blood results, policies for thrombosis prevention and preventing avoidable blood transfusion. In response, the American Society of Haematology (ASH) formally adopted the term Classical Haematology to affirm the importance of these conditions.^3^ While this initiative has been valuable in strengthening training pathways in the United States,^4^ the term ‘Classical’ may not fully capture the dynamism of modern science and therapeutics driving the field.^5^ It has also gained limited institutional recognition and policy resonance in many international settings, including the United Kingdom, Europe and low‐ and middle‐income countries (LMICs). We therefore propose a complementary framework and advocate for the formal adoption of the term Medical Haematology to describe this broad spectrum of haematology that directly underpins and integrates with general medicine, surgery, obstetrics and critical care. The adjective medical is used not in opposition to Haemato‐Oncology, which remains a medical discipline in its own right but to position this field firmly within the continuum of patient‐centred medical care.

The purpose of this paper in advocating use of the term Medical Haematology is twofold. First, to articulate why reclaiming the clinical and integrative identity of haematology is essential to the sustainability of the specialty. Second, to outline actionable strategies, for example, through education, workforce reform and global health leadership, which can re‐establish haematology's position at the heart of modern medicine. By redefining the field in medical terms, we aim to align it more closely with national health priorities, attract a new generation of clinicians and address inequities in research and global health outcomes.

## THE FOUNDATIONS OF HAEMATOLOGY IN MEDICINE

The origins of haematology are deeply rooted in the earliest medical practices. Bloodletting, once a mainstay of treatment, reflected ancient beliefs about blood's vital role in health.^6^ The field has repeatedly defined new paradigms in diagnosis, pathophysiology and translational science. Table 1 summarises key milestones that illustrate how discoveries in blood have continually reshaped the wider practice of medicine. These advances chart an unbroken continuum from microscopy to molecular medicine. The introduction of the microscope in the 17th century confirmed the cellular nature of blood and laid the foundations of morphology that remain vital for global health diagnostics today.^7^, ^8^ The discovery of the ABO and Rh blood group systems by Landsteiner and colleagues at the turn of the 20th century made transfusion safe and enabled modern surgery and obstetric care.^9^ Whipple's demonstration that liver‐reversed pernicious anaemia established a template for targeted metabolic therapy and exemplified the ability of haematology to translate bench discoveries directly into lifesaving treatment.^10^

**TABLE 1:** Foundational discoveries of haematology in medicine.

Domain	Year of milestone	Discovery/innovation	Impact on medicine
Diagnostics	1674	A. van Leeuwenhoek describes human red blood cells	Confirmed the cellular nature of blood, founding haematological morphology
	1770s–1820s	W. Hewson describes fibrin, coagulation and identifies white blood cells	Established the core components of blood and the basis of its clotting function
	1840s–1860s	O. Funke, E. Hoppe‐Seyler and F. Hünefeld isolate and characterise haemoglobin	Identified the oxygen‐carrying pigment of blood, a cornerstone of physiology
	1877	P. Ehrlich develops blood cell staining techniques	Enabled differentiation of leucocytes and diagnosis of blood diseases
Therapeutics	1934	G. Whipple, G Minot and W Murphy discover liver therapy for pernicious anaemia	First successful targeted nutrient therapy for a vitamin deficiency; a Nobel Prize‐winning milestone
Anticoagulation	1916–1930s	Discovery of heparin (McLean, Howell) and vitamin K antagonists	Provided the first effective pharmacological tools to prevent and treat thrombosis
	2000s	Development of direct oral anticoagulants (DOACs)	Created a more convenient class of drugs for long‐term anticoagulation
Fibrinolysis & haemostasis	1934	W.S. Tillett discovers the fibrinolytic agent streptokinase	Laid the groundwork for clot‐dissolving (thrombolytic) therapy
	1947–52	T. Astrup and P.M. Permin identify and characterise tissue‐type plasminogen activator (tPA)	Discovered the key physiological agent for targeted fibrinolysis
	1962	S. and U. Okamoto discover the antifibrinolytic agent tranexamic acid	Provided a potent tool to inhibit pathological fibrinolysis and reduce bleeding
Immunology & transfusion	1890s	E. Metchnikoff discovers phagocytosis by white blood cells	Pioneered immunohaematology and the concept of cellular host defence
	1900	K. Landsteiner discovers ABO blood groups	Made safe blood transfusion possible—prerequisite for modern surgery
	1937	P. Levine & R. Stetson identify the Rhesus system	Improved transfusion safety and understanding of maternal–fetal compatibility
Molecular medicine	1949	L. Pauling characterises sickle haemoglobin	Pioneered the concept of ‘molecular disease’
	1959	Nowell & Hungerford identify the Philadelphia chromosome	First link between a chromosomal abnormality and cancer (CML)
Targeted therapy	2001	Imatinib (tyrosine kinase inhibitor) approved for CML	Ushered in the era of precision oncology
Immunotherapy	1997	Rituximab (anti‐CD20 monoclonal antibody) approved for NHL	Pioneered monoclonal antibody therapy, now used across disciplines
	2017	CAR‐T cells approved for leukaemia/lymphoma	A new pillar of cancer and autoimmune treatment
Gene therapy	2022–2023	CRISPR/Cas9 and gene therapy approved for sickle cell disease, β‐thalassaemia and haemophilia B	A transformative therapeutic modality pioneered for genetic diseases

*Note*: A chronology of seminal discoveries originating in haematology that have provided foundational concepts, diagnostic tools and therapeutic modalities for modern medicine, underscoring the speciality's central and integrative role.

Abbreviations: CAR‐T, chimeric antigen receptor T‐cell therapy; CML, chronic myeloid leukaemia; CRISPR‐Cas9, Clustered Regularly Interspaced Short Palindromic Repeats (CRISPR) and CRISPR‐associated protein 9; NHL, non‐Hodgkin lymphoma.

Mid‐20th‐century breakthroughs cemented the reputation of haematology as a driver of biomedical innovation. The characterisation of sickle haemoglobin introduced the concept of the molecular disease^11^ and the identification of the Philadelphia chromosome linked chromosomal abnormality to malignancy for the first time.^12^ These insights heralded the molecular revolution that transformed both Haemato‐Oncology and Medical Haematology. Subsequent advances, from the development of tyrosine‐kinase inhibitors and monoclonal antibodies to chimeric antigen receptor T‐cell therapy, show how haematological science continues to pioneer modalities later adopted across all of medicine.^13^

Haematology has also led in immunology and host defence. In addition to underpinning safe transfusion, advances include the use of immunoglobulins in autoimmune cytopenias and the evolution of targeted therapies such as anti‐CD20 antibodies and complement inhibitors.^14^, ^15^ The speciality's translational agility was again evident during the COVID‐19 pandemic, when haematologists defined the pathophysiology of vaccine‐induced immune thrombocytopenia and thrombosis (VITT) and rapidly developed effective treatment protocols.^16^ These examples reinforce the unique interface of haematology between immunity, inflammation and vascular biology.

Gene and cell therapies represent the latest chapter in this trajectory. The approval of gene editing for sickle cell disease, β‐thalassaemia and haemophilia B demonstrates how Medical Haematology continues to deliver first‐in‐class interventions for inherited disorders while providing a framework for applying genomic medicine across specialities.^17^, ^18^

Together, these milestones show that haematology has never been a peripheral science. It has consistently anticipated new frontiers—from microscopy to molecular genetics—and continues to anchor medical progress. The same capacity for innovation can now underpin its renewed clinical identity as Medical Haematology, bridging the laboratory and bedside to deliver integrated care for patients worldwide.

## MEDICAL HAEMATOLOGY IN PRACTICE

The reach from Haematology extends far beyond the laboratory. As a clinical discipline, it provides essential input across cardiology, obstetrics, gastroenterology, nephrology, intensive care and surgery. Few specialities exert such pervasive influence on patient outcomes, largely through the prevention and management of bleeding and thrombosis, rational transfusion and diagnosis of cytopenic and immune‐mediated disorders (Table 2).

**TABLE 2:** Examples of scope and impact of medical haematology.

Clinical discipline	Key haematological challenges	Role of the medical haematologist
Cardiology & stroke medicine	Anticoagulation in AF; stroke prevention	Personalise antithrombotic therapy; diagnose and manage prothrombotic states
Obstetrics & gynaecology	High‐risk pregnancy (thrombophilia, ITP); antiphospholipid syndrome; postpartum haemorrhage	Manage anticoagulation; guides peripartum care for bleeding and clotting disorders
Gastroenterology	Increased risk of VTE in IBD	Lead thromboprophylaxis protocols and risk assessment
Nephrology	Anaemia of chronic kidney disease	Optimise iron therapy and erythropoiesis‐stimulating agents to reduce transfusion dependence
Intensive care & infectious diseases	Sepsis‐induced DIC; immunothrombosis	Diagnose and manage life‐threatening coagulopathies; advise on transfusion and plasma exchange
Surgery & perioperative care	Bleeding risk; thrombosis prevention; patient blood management	Optimise transfusion and correct preoperative anaemia
Oncology (solid tumours)	Cancer‐associated thrombosis; chemotherapy‐induced cytopenias	Partner in managing thrombotic complications and treatment‐related cytopenias
Primary care	Anaemia screening; thrombophilia risk; drug‐induced cytopenias	Ensure continuity of anticoagulation care and safe prescribing in the community
Public & global health	Anaemia; haemoglobinopathies	Shape national policy, prevention strategies in LMICs

*Note*: Representative examples of how Medical Haematology contributes to major clinical and public‐health domains.

Abbreviations: AF, atrial fibrillation; APS, antiphospholipid syndrome; DIC, disseminated intravascular coagulation; IBD, inflammatory bowel disease; ITP, immune thrombocytopenia; LMICs, low‐ and middle‐income countries; VTE, venous thromboembolism.

In cardiology and stroke medicine, haematologists refine anticoagulation strategies for atrial fibrillation and antiphospholipid syndrome, balancing thrombotic and bleeding risk.^19^ In obstetrics, they guide management of thrombophilia, immune thrombocytopenia and postpartum haemorrhage—conditions in which prompt haematological input is lifesaving.^20^ Gastroenterology and inflammatory bowel disease illustrate the systemic relevance of thrombosis prevention,^21^ while in nephrology, expertise in iron therapy and erythropoiesis‐stimulating agents reduces transfusion dependence in chronic kidney disease.^22^ Collaboration with oncology extends to the management of cancer‐associated thrombosis and treatment‐related cytopenias, ensuring safe delivery of systemic therapy.^23^ Increasingly, critical care settings highlight the speciality's central role in managing sepsis‐induced coagulopathy and immune thrombosis, including COVID‐19‐related disorders and VITT.^24^, ^25^ In perioperative and surgical care, haematologists lead multidisciplinary teams that optimise transfusion, correct preoperative anaemia and prevent venous thromboembolism.

However, this demand for haematology input across patient care in hospitals is increasing with evidence from national comparative audits showing that investigation and management remain suboptimal. This is especially evident in the perioperative setting where the underuse of proven alternatives to transfusion, such as the preoperative correction of iron deficiency and the systematic administration of tranexamic acid, represents a major gap in care.^26^ This failure to optimise patient blood management has serious consequences; data from the Serious Hazards of Transfusion scheme indicate that transfusion‐related mortality in the United Kingdom is now four times higher than it was a decade ago.^27^, ^28^ This stark trend underscores the urgent need for haematologists to drive the implementation of safer, more effective strategies, including standardised investigation pathways and targeted treatments for at‐risk groups.^29^

Altogether, these roles demonstrate that Medical Haematology is not a niche sub‐speciality but a clinical partner embedded across medicine and surgery. Its breadth and integrative perspective place it uniquely to improve patient safety, steward finite resources and advance equity.

## HAEMATOLOGY IN GLOBAL HEALTH AND POPULATION MEDICINE

The global health footprint of Medical Haematology mirrors the themes of earlier sections: a discipline whose foundational science translates directly into population‐level impact. Just as haematology has shaped molecular medicine and everyday clinical practice, it is also central to addressing some of the world's most pervasive and inequitable health burdens. Anaemia remains the clearest example. In England, anaemia affects approximately 1 in 25 people, with 2019 prevalence data showing rates of 4.1% in women and 3.1% in men.^30^ The condition is more common in older adults, people of Black and Asian ethnicities and those living in areas of higher social deprivation. Worldwide, an estimated 1.92 billion people are affected, with prevalence exceeding 40%–50% among women and young children in sub‐Saharan Africa and South Asia.^31^, ^32^ Its consequences extend well beyond fatigue and pallor: It is responsible for more than 50 million years lived with disability, contributes substantially to maternal mortality—around 20‐fold higher in low‐income countries—and impairs child development and economic productivity.^33^, ^34^ Anaemia of inflammation (formerly termed anaemia of chronic disease) is also increasing in high‐income settings as populations age, reflecting multimorbidity across heart failure, chronic kidney disease, diabetes and inflammatory states.^35^ These patterns collectively demonstrate why anaemia cannot be viewed as a ‘benign’ or secondary concern and why it demands coordinated haematological leadership across health systems.

Inherited haemoglobin disorders further emphasise the need for structured haematological expertise. LMICs shoulder 80% of the global haemoglobinopathy burden, with 300 000–500 000 children born annually with sickle cell disease or thalassaemia.^36^ Prevention strategies, newborn screening and access to disease‐modifying therapies remain inconsistent, underscoring the need for haematologists to shape national policies, training and implementation programmes.

Thrombosis is an equally urgent challenge. Venous thromboembolism causes more than 300 000 deaths annually in Europe, exceeding mortality from breast and prostate cancer combined. Sixty per cent of events are hospital‐associated.^37^, ^38^ Yet access to anticoagulation remains profoundly uneven. Cost and regulatory barriers limit the availability of direct oral anticoagulants in many LMICs,^39^ although successful programmes in Africa and South Asia demonstrate that safe anticoagulation is achievable and scalable when led by haematologists.^40^, ^41^

Acute haemorrhage control offers some of the most compelling examples of haematology's population‐level impact. Postpartum haemorrhage kills 70 000 women annually, despite strong evidence from the WOMAN trial that early tranexamic acid reduces mortality by one‐third.^42^ Similar gaps exist in trauma care, where underuse of tranexamic acid persists despite robust evidence from the CRASH‐2 and CRASH‐3 trials.^43^ These implementation failings highlight the need for haematologists to lead guideline development, advocacy for essential medicines and transfusion system strengthening—an urgency reinforced by findings of the UK Infected Blood Inquiry.^44^

These examples show that Medical Haematology is indispensable to global health—one that aligns with national health strategies, World Health Organization (WHO)‐endorsed approaches to patient blood management and the lived realities of systems facing the greatest haematological burden.

## THE CASE FOR MEDICAL HAEMATOLOGY

As the preceding sections demonstrate the field's vast clinical, scientific and global health footprint, the current nomenclature of ‘non‐malignant haematology’ is both semantically and substantively flawed. By defining the field through negation, it implicitly devalues its scope and significance, despite encompassing conditions that are complex, life‐threatening and integral to patient care. This misnomer has tangible and damaging consequences across multiple domains.^45^, ^46^

Patient safety is compromised when disorders with a high mortality, such as sickle cell disease and TTP, are labelled ‘benign’ or ‘non‐malignant’. As argued by Al‐Samkari et al., such terminology belies the severity of these conditions and risks trivialising the experiences of affected individuals.^47^ Rebranding is not merely symbolic but a necessary step towards affirming the gravity of these diseases and the expertise required to manage them.

Workforce recruitment and training are critically impacted by incorrect terminology. In the United States, only 4%–5% of haematology trainees express interest in non‐malignant disciplines, while surveys in Europe reveal significant gaps in clinician confidence in areas such as haemostasis, thrombosis, obstetric haematology and autoimmune haematological disorders.^48^, ^49^ This shortage occurs amid rising clinical demand, particularly with the increasing burden of chronic diseases such as anaemia of inflammation—the second most common anaemia globally, which is strongly linked to multimorbidity in ageing populations.^50^, ^51^ The persistence of an unattractive and ill‐defined professional identity exacerbates these recruitment challenges.

Research funding disparities further marginalise the field. Currently, 85% of haematology research investment targets malignant conditions, despite non‐malignant disorders affecting billions worldwide.^52^, ^53^ Anaemia alone impacts 2 billion people yet remains profoundly understudied. Increasingly, genetic links between haematological pathways and common non‐communicable diseases are being uncovered, highlighting opportunities for scientific advancement that are currently neglected due to branding and prioritisation failures.

The term Classical Haematology, as proposed by Al‐Samkari and colleagues, offers a solution.^46^ It rightly honours the historical roots of the field and avoids the pejorative connotations of ‘benign’ or ‘non‐malignant’. However, we propose that ‘Medical Haematology’ offers a more clinically integrative identity for the future. While ‘classical’ evokes a foundational and traditional past, ‘medical’ explicitly conveys a patient‐centred, front‐line and holistic practice that is central to modern healthcare systems. It aligns with the reality of the haematologist's role as a physician managing complex inpatients, leading multidisciplinary teams and stewarding hospital‐wide resources like anticoagulation and transfusion. This terminology reinforces the speciality's outward‐looking collaboration with other medical disciplines, from obstetrics to intensive care, rather than primarily defining itself in relation to its historical roots.

Embracing ‘Medical Haematology’ is not a rejection of the past or a competing label but complements by providing a health system‐neutral and internationally recognisable identity that is particularly relevant to the United Kingdom, Europe and LMIC. It is both an affirmation of the speciality's evolving role at the forefront of hospital medicine and a strategic imperative to secure the workforce, research investment and clinical influence that these vital disciplines demand and deserve. The opportunity to be involved in such a wide diversity of practice should also be highly attractive to early‐career haematologists.

## THE FUTURE OF MEDICAL HAEMATOLOGY

**FIGURE 1:**
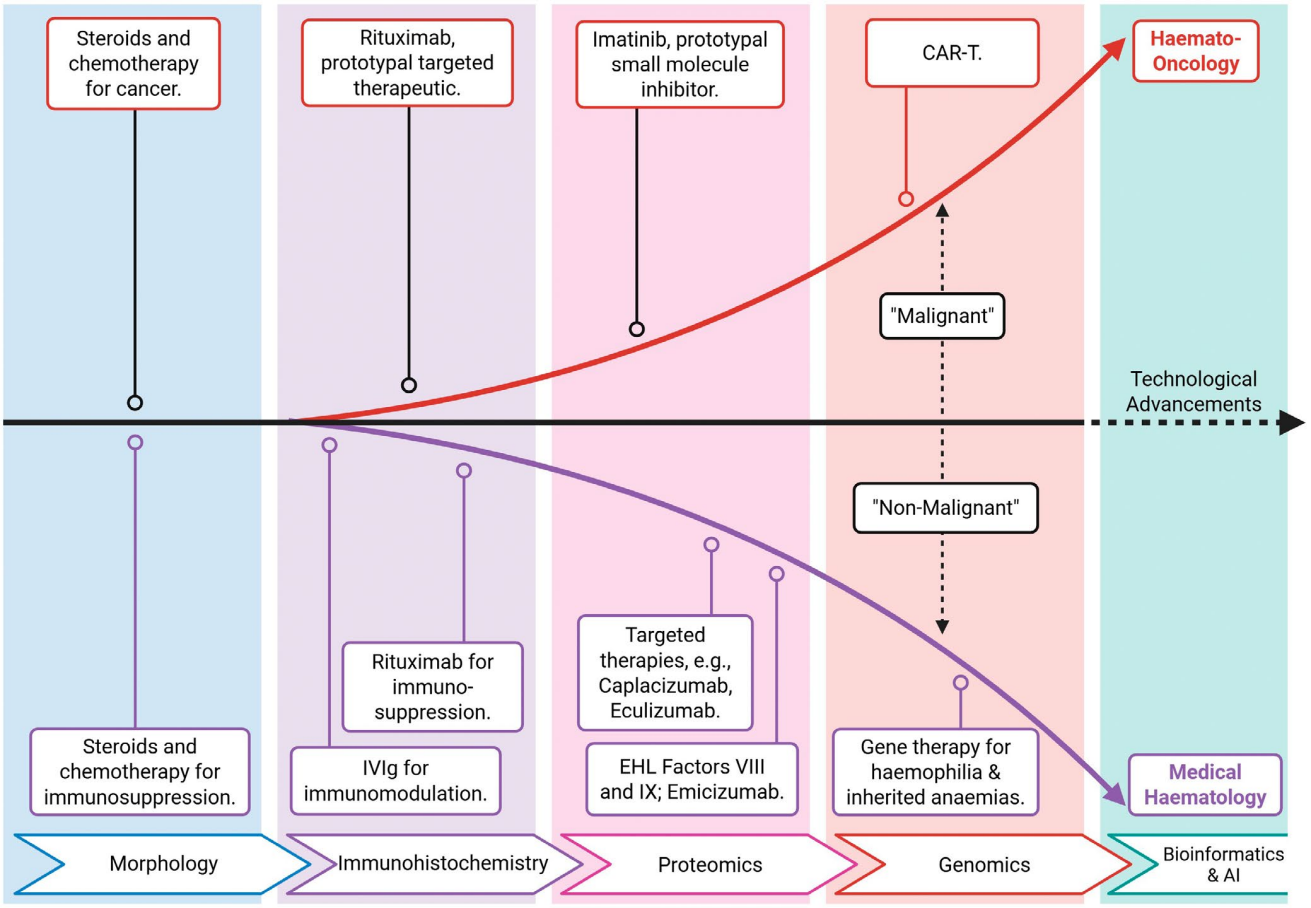
Medical haematology and haematology‐oncology—key milestones in haematology diagnostics and treatments used across medicine. These advancements generated a divide into the broad categories of ‘malignant’ and ‘non‐malignant’ haematology. CAR‐T, chimeric antigen receptor T cell; EHL, extended half‐life.

Medical Haematology is poised at a transformative juncture with the prospect of harnessing cutting‐edge genomic and computational innovations to redefine patient care (Figure 1). Genome‐wide association studies (GWAS) have proven to be a powerful tool for identifying genetic variants associated with various diseases.^54^ While initially focused on diseases like coronary artery disease (CAD) and hypertension, where blood and vascular systems are directly implicated, GWAS has also uncovered unexpected connections between these systems and seemingly unrelated conditions. These include Crohn's disease, where genes involved in the cellular process of autophagy are also involved in blood vessel function and point to a possible link between vascular health and inflammatory bowel disease.^55^ GWAS meta‐analyses of UK Biobank have also identified shared genetic pathways between insulin resistance and CAD, with haematology‐related genes (CCDC92, ARHGEF26) influencing endothelial function.^56^ This highlights the close relationship between metabolic and vascular health and the potential for shared genetic pathways.

In spontaneous coronary artery dissection (SCAD), a *Nature Genetics* publication identified 16 risk loci linking arterial integrity and tissue‐mediated coagulation with *F3* (tissue factor gene) emerging as a SCAD‐specific risk variant.^56^ These findings underscore haematology's role in vascular biology beyond traditional clotting disorders, which will be increasingly relevant in an ageing society. In addition, GWAS of blood cell morphology revealed 2172 variant–trait associations, implicating genes like *IL2RA* and *ITGA4* in autoimmune diseases—mirroring drug targets (e.g. daclizumab for multiple sclerosis).^57^ Such discoveries blur boundaries between haematology, immunology and neurology.

The integration of GWAS data with artificial intelligence (AI) is accelerating breakthroughs beyond unlocking the blood‐vascular gene networks.^58^ Soon, scientists will be able to mine massive population biobanks that are currently being curated, which will combine genome‐wide data with detailed information on measured traits, lifestyle, diet and environmental exposures.^59^, ^60^ These will be tools that will hasten the decades‐long shift from haematologists being laboratory pathologists only towards repositioning and recognition as front‐line care physicians.^61^ It will elevate the speciality's role in multidisciplinary collaboration and precision medicine to reinforce the centrality of Medical Haematology in system‐wide health globally.

In diagnostics, deep learning models will be able to interpret blood films with pathologist level accuracy. The prospect of AI‐driven platforms predicting risks of adverse events to enable pre‐emptive intervention is also real, especially with improvements in GWAS‐derived polygenic risk scores and electronic health records.^62^ With increasing emphasis on data from diverse populations that have already uncovered ancestry‐specific variants, edge‐computing devices can bring haematology to resource‐limited settings, potentially bridging gaps in sickle cell care.

## FRAMEWORK AND ACTION PLAN FOR MEDICAL HAEMATOLOGY

The revitalisation of Medical Haematology requires strategic leadership and coordinated action across education, clinical service delivery, research prioritisation and global health equity. Although cognate with ‘Medical Oncology’, this parallel is instructive. It delineates a distinct speciality within the broader haematology discipline, just as medical oncology is distinct from surgical or radiation oncology. This clarity is useful for trainees, patients and healthcare systems in understanding the scope of practice. We therefore propose five interlocking strategies to secure the speciality's future. They provide a coherent framework that aligns with national health priorities, workforce pressures and the global burden of haematological disorders.

### Establish ‘Blood Teams’ as a core hospital service

Haematology should be embedded as a proactive clinical service rather than a reactive consultative speciality. We propose the formal establishment of Blood Teams—multidisciplinary groups led by haematologists and including transfusion practitioners, pharmacists and specialist nurses—to conduct daily or virtual ward rounds focused on anticoagulation, transfusion, haemostasis and anaemia management. This model mirrors the success of antimicrobial stewardship and has already shown promise in several United Kingdom and international centres. Benefits include reduced inappropriate transfusion, improved anticoagulation safety, shorter length of stay and enhanced compliance with national guidelines. Pilot programmes could define scalable metrics for length of stay, venous thromboembolic events, transfusion utilisation and avoidable harm, providing the evidence base needed for health‐system adoption.

### Secure and rebuild the workforce

Workforce shortages represent the single greatest threat to the sustainability of Medical Haematology. In the United Kingdom, the number of medical trainees within haematology departments has fallen by more than one‐third (36%) in recent years, and surveys across Europe and North America consistently demonstrate declining interest among trainees, insufficient exposure to non‐malignant disciplines and a widening mismatch between clinical demand and consultant capacity.^63^, ^64^ Rising multimorbidity, ageing populations and expanding responsibilities in thrombosis, transfusion, obstetrics, perioperative medicine and global health further compound these pressures.^65^ Importantly, National Health Service and European Union regulatory frameworks explicitly use ‘medical specialties’ as formal organisational categories; consequently, framing this domain as Medical Haematology aligns more naturally with these systems and strengthens workforce planning, service design and policy dialogue in a way that the term Classical Haematology does not. A coherent workforce strategy should include:

#### Strengthening identity and visibility

A clear, clinically resonant identity is essential for attracting trainees who seek broad‐based, acute medical careers. Terminology is important, including in LMICs, where terms aligned with service delivery and patient care carry far greater relevance for policymakers, training bodies and early‐career clinicians.

#### Restoring balance within training programmes

To rebalance the current under‐representation of Medical Haematology in training programmes,^66^ there should be:

- equivalent exposure to thrombosis, haemostasis, transfusion, obstetrics, immune cytopenias and global health;
- joint rotations with cardiology, obstetrics, intensive care, nephrology and emergency medicine; and
- protected time for academic development in medical interfacing areas that include implementation science, genomics and health‐systems research.

#### Creating attractive and flexible career pathways

To improve recruitment and retention:

- develop combined posts (e.g. Haematology and General Medicine, Haematology and Critical Care);
- expand consultant job plans to recognise the value of hospital‐wide leadership roles (e.g. anticoagulation, transfusion, thrombosis committees); and
- implement mentorship networks via the British Society for Haematology (BSH), European Hematology Association (EHA) and ASH, with a focus on women, minority groups and clinicians in LMICs.

#### Building global capacity

In LMICs, which often face the most acute specialist shortages, Medical Haematology offers a framework that resonates with clinical need and can underpin:

- regional training hubs in Africa, South Asia and the Middle East;
- twinning programmes and short‐term specialist exchanges; and
- standardised, competency‐based curricula aligned with national health targets and the WHO patient blood management principles.

A strengthened, internationally relevant workforce is fundamental to advancing patient safety and equity.

### Modernise training and education

Current under‐ and postgraduate curricula under‐represent the breadth of Medical Haematology. Reform should begin early in medical school and extend through to consultant accreditation.^67^ Key actions include:

- integrating Medical Haematology teaching within internal medicine and acute care rotations;
- developing (inter)national case‐based teaching modules on venous thromboembolism, transfusion, immune cytopenias, obstetric bleeding and therapeutics^68^;
- embedding genomic literacy, bioinformatics and AI‐enabled diagnostics of both peripheral blood and bone marrow into core training^69^; and
- expanding mentorship, supervision and structured exposure to non‐malignant specialties.

This requires collaboration between the BSH, UK Royal Medical Colleges, EHA, ASH and national training bodies to standardise competencies and support exchange programmes, ensuring global parity in training.

### Champion research equity and global health

Despite non‐malignant disorders affecting billions, the vast majority of haematology research funding remains directed towards malignancy.^52^ Medical Haematology must advocate for:

- dedicated funding streams for anaemia, thrombosis, haemostasis, obstetric haematology and haemoglobinopathies;
- implementation research and real‐world evaluation, particularly in data‐driven care in transfusion and thrombosis;
- collaborative global registries for sickle cell disease, TTP and autoimmune cytopenias; and
- access to essential diagnostics (full blood count, ferritin, coagulation profiles) and treatments (tranexamic acid, direct oral anticoagulants).

Embedding global health within national research agendas will help bridge the translational gap for populations most affected by haematological disease.

### Embrace genomic and computational innovation

The molecular heritage of haematology positions the speciality to lead in integrating genomics, bioinformatics and AI‐driven diagnostics. Future priorities include:

- training clinicians to interpret polygenic risk scores, genomic reports and real‐time biomarker data;
- establishing partnerships with national biobanks to ensure diverse population representation;
- deploying edge‐computing diagnostics and AI‐assisted smear interpretation for resource‐limited settings; and
- ensuring technological advances deliver practical, equitable improvements in patient outcomes.

Together, these actions form a pragmatic and forward‐looking blueprint for strengthening the visibility, capacity, sustainability and global relevance of the speciality.

## CONCLUSION

Haematology stands at a critical juncture. The path of increasing fragmentation, with its attendant workforce crises and research inequities, is unsustainable. The alternative and conscious embrace of Medical Haematology offers a route to reassert its universal relevance in modern medicine. This is more than a name change; it is a strategic recalibration. Medical Haematology complements the principles of Classical Haematology by anchoring them in the realities of health‐system design and multidisciplinary care. The term situates the haematologist alongside cardiologists, nephrologists, intensivists and obstetricians as a physician who bridges molecular mechanisms and patient outcomes.^70^, ^71^ It asserts that haematology is not a peripheral consultancy but a clinical discipline indispensable to diagnosis, prevention and therapeutics across the entire spectrum of hospital and community medicine.

Ultimately, embracing Medical Haematology is not divisive. Fundamentally, it projects a future defined by collaboration, equity and precision medicine that is globally resonant. By reclaiming its position at the heart of clinical care, haematology can again be recognised for what it has always been—a discipline that illuminates the mechanisms of life, unites science and practice and saves lives across many corners of medicine.

## AUTHOR CONTRIBUTIONS

CHT contributed to the conceptualisation, literature search, writing of the original draft, reviewing, editing and final approval of the manuscript. IB and SP contributed to the literature search, reviewing, editing and final approval of the manuscript.

## CONFLICT OF INTEREST STATEMENT

CHT is Chair of the National Blood Transfusion Committee and a Past‐President of the British Society for Haematology. SP is the current President of the British Society for Haematology.

## Data Availability

Data sharing not applicable to this article as no datasets were generated or analysed during the current study.
